# 288 Antimicrobial Prescribing Patterns in Dogs and Cats at a Michigan Veterinary Medical Center

**DOI:** 10.1017/ash.2026.10647

**Published:** 2026-06-23

**Authors:** Jordan Cates, Laura Calderwood, Aaron Curns, Sara Mirza

**Affiliations:** 1 CDC; 2 Cherokee Nation Operational Solutions and Division of Viral Diseases, CDC; 3 U.S. Centers for Disease Control and Prevention

## Abstract

**Background:** Long-term care facilities (LTCFs) are a leading setting of norovirus acute gastroenteritis (AGE) outbreaks in the United States. While illness from norovirus infection is typically considered mild and self-limiting, residents in LTCFs are especially vulnerable to more severe outcomes such as hospitalization and, in rare instances, death. Norovirus typically spreads rapidly through LTCFs due to the high infectivity of the virus and the congregate nature of these facilities. Despite the high burden of norovirus outbreaks in the U.S., limited data exist in these settings. Our objective was to characterize clusters of AGE likely associated with a norovirus outbreak among LTCF residents in a large cohort of U.S. LTCFs. **Methods:** We analyzed electronic health records from the PointClickCare (PCC) database of residents in 14,588 LTCFs across 50 states, D.C., and 1 US territory from 2016 through 2023. We identified AGE episodes using ICD-10-CM diagnosis codes and defined AGE clusters as three or more residents of the same facility with AGE episodes within seven days of one another; norovirus-associated AGE clusters were defined as clusters with at least one norovirus or unspecified viral intestinal infection ICD-10-CM code (A08.1, A08.4). **Results:** From January 1, 2016 through December 31, 2023, there were 306,683 AGE episodes from 10,653 LTCFs. Among these LTCFs, there were 17,852 AGE clusters of 86,687 (29%) episodes; 648 were norovirus-associated AGE clusters comprising 3,548 AGE episodes. The median norovirus-associated AGE cluster size was 4 residents (interquartile range [IQR]) 3-6; maximum=36). The number of norovirus-associated AGE clusters was lowest in June, July, August, September, and October (average of 3 clusters each month) and was highest in February and March (average of 13 clusters each month). The median (IQR) length of norovirus-associated AGE clusters was 9 (6-13) days from the AGE onset of the first resident to the AGE onset of the last resident in the cluster. Norovirus-associated clusters occurred in 492 facilities: 141 (29%) facilities of <100 residents, 288 (58%) facilities of 100-199 residents, and 63 (13%) facilities of 200+ residents. **Conclusions:** AGE episodes were commonly reported among LTCF residents in PCC, and approximately 30% were associated with an AGE cluster. Norovirus-associated AGE clusters ranged in size, occurred in all sizes of facilities, and were more common in winter months. Identifying individual-level and facility-level risk factors for norovirus-associated AGE is a next step to inform prevention efforts, including infection control measures and target populations for future norovirus vaccines.

